# Essential Oils Composition and Antioxidant Properties of Three *Thymus* Species

**DOI:** 10.1155/2012/728065

**Published:** 2011-08-25

**Authors:** Hamzeh Amiri

**Affiliations:** Department of Biology, Lorestan University, P.O. Box 465, Khoram-Abad, Iran

## Abstract

The essential oils of three wild-growing *Thymus* species, collected from west of Iran during the flowering stage, were obtained by hydrodistillation and analyzed by gas chromatography (GC) and gas chromatography/mass spectrometry (GC-MS). Under the optimum extraction and analysis conditions, 44, 38, and 38 constituents (mainly monoterpenes compounds) were identified in *T. kotschyanus* Boiss. and Hohen, *T. eriocalyx* (Ronniger) Jalas, and *T. daenensis subsp lancifolius* (Celak) Jalas which represented 89.9%, 99.7%, and 95.8% of the oils, respectively. The main constituents were thymol (16.4–42.6%), carvacrol (7.6–52.3%), and *γ*-terpinene (3–11.4%). Antioxidant activity was employed by two complementary test systems, namely, 2,2-diphenyl-1-picrylhydrazyl (DPPH) free-radical scavenging and *β*-carotene/linoleic acid systems. Antioxidant activity of polar subfraction of *T. daenensis subsp lancifolius* (Celak) Jalas was found to be higher than those of the others in DPPH assay, while nonpolar subfraction of *T. eriocalyx* (Ronniger) Jalas has most antioxidant activity in *β*-carotene/linoleic acid test (19.1 ± 0.1 *μ*g/mL and 96.1 ± 0.8% inhibition rate, resp.).

## 1. Introduction

Reactive oxygen species (ROS) including singlet oxygen (^1^O^2^), superoxide ion (O_2_^−·^), hydroxyl ion (OH^*·*^), and hydrogen peroxide (H_2_O_2_) are highly reactive and toxic molecules generated in cells under normal metabolic activities. However, in response to a variety of factors including tobacco smoke, pollutants, ionising radiations, alcohol, synthetic pesticides, and solvent, their production increases [12]. ROS can cause oxidative damage to proteins, lipids, enzymes, and DNA, and they have also been linked to pathogenesis of oxidative diseases [13]. Living cells possess an excellent scavenging mechanism to avoid excess ROS-induced cellular injury; however, with ageing and under influence of external stresses, these mechanisms become inefficient, and dietary supplementation of synthetic antioxidants is required. In recent years, due to toxicological concerns associated with the use of synthetic substances in food and increasing awareness about natural foods, there has been an increased interest in the use of natural substances as food preservatives and antioxidants [14]. In this context, aromatic plants, particularly their essential oils, are being evaluated for antioxidant activity. It is, thus, pertinent to evaluate the natural antioxidant activity of essential oils, since they find extensive use in the food and beverage industry [15–17]. Many natural molecules, especially those produced in the plant kingdom, have at least one benzene ring with a hydroxyl functional group in their skeleton. Such compounds are collectively known as phenolic compounds and, due to their hydrogen or single electron donating potentials, usually play important rules in the antioxidant activity of the plant extracts [18].

The genus *Thymus* L., known as “Avishan” in Persian, is a well-known aromatic perennial herb originated from Mediterranean region. Among 215 species of this genus grown in the world, 14 species are distributed in Iranian flora [19]. *Thymus* species are well known as medicinal plants because of their biological and pharmacological properties. In traditional medicine, leaves and flowering parts of *Thymus* species are widely used as tonic and herbal tea, antiseptic, antitussive, and carminative as well as treating colds [20, 21]. *Thymus* oils and extracts are widely used in pharmaceutical, cosmetic, and perfume industry, also for flavoring and preservation of several food products [22].

In this study, antioxidant tests carried out by two complementary test systems, namely, 2,2-diphenyl-1-picrylhydrazyl (DPPH) free-radical scavenging and *β*-carotene/linoleic acid systems. DPPH is the best, easiest and widely used method for testing preliminary free-radical scavenging activity of a compound or a plant extract. The effects of antioxidants in the DPPH-radical-scavenging test reflect the hydrogen-donating capacity of a compound. When the radical form of DPPH is scavenged by an antioxidant through the donation of a hydrogen to form a stable DPPH molecule, this leads to a colour change from purple to yellow, and a decrease in absorbance [23]. In the *β*-carotene bleaching test, *β*-carotene undergoes rapid discoloration in the absence of an antioxidant. This is because the coupled oxidation of *β*-carotene and linoleic acid generates free radicals. The linoleic acid free radical formed upon the abstraction of a hydrogen atom from one of its diallylic methylene groups attacks the highly unsaturated *β*-carotene molecule. As a result, *β*-carotene is oxidized and breaks down in part, subsequently losing its chromophore and characteristic orange colour, which is monitored spectrophotometrically [24].

The aim of this study is to evaluate the chemical composition of essential oils and *in vitro* antioxidant activity of the essential oils and methanol extracts of *T. kotschyanus*, *T. eriocalyx,* and *T. daenensis subsp lancifolius*.

## 2. Materials and Methods

### 2.1. Plant Material

The aerial parts of *T. kotschyanus* Boiss. and Hohen, *T. eriocalyx* (Ronniger) Jalas and *T. daenensis subsp lancifolius* (Celak) Jalas were collected during flowering stage (10th Jun to 15th Aug 2009) from Lorestan Province, Southwest of Iran. Voucher specimens (no: 7384) were identified by Dr. Mohammad Mehrnia and have been deposited at the Herbarium of Agriculture and Natural Resources Center of Lorestan Province, Khoramabad, Iran. Collected plant materials were dried in the shade.

### 2.2. Isolation of the Essential Oils

Dry aerial parts (100 g) of *T. kotschyanus*, *T. eriocalyx* and *T. daenensis subsp lancifolius* were subjected to the hydrodistillation of 2.5 h, using a cleavenger-type apparatus, according to the method recommended by the European Pharmacopia [25] to produce oils. The obtained essential oils were dried over anhydrous sodium sulphate and stored at +4°C until tested and analysed.

### 2.3. Preparation of Methanol Extracts

The air-dried and finely ground samples were extracted by using the method described previously [26]. Briefly, the sample weighing about 100 g were extracted in a Soxhlet with methanol (MeOH) at 60°C for 6 h. The extracts were then filtered and concentrated *in vacuo* at 45°C yielding a waxy material. The resulting extracts were suspended in water and partitioned with chloroform (CHCI_3_) to obtain water-soluble (polar) and water-insoluble (nonpolar, chloroformic) subfractions. Extracts were concentrated, dried, and kept in the dark at +4°C until tested.

### 2.4. Analysis of the Oils

FID-GC was carried out using a Hewlett-Packard 6890 with DB-5 capillary column (phenyl methyl diloxane, 25 m × 0.25 mm i.d., 0.25 *μ*m film thickness); carrier gas, He; split ratio, 1 : 25, and flam ionization detector. Temperature progamme: 60°C (2 min) rising to 240°C at 4°C/min, injector temperature, 250°C, detector temperature, 260°C. GC-MS was performed using Hewlett-Packard 6859 with quadrupole detector, on a HP-5 column (see GC), operating at 70 eV ionization energy, using the same temperature programme and carrier gas as above. Retention indices were calculated by using retention times of *n*-alkanes that were injected after the oils at the same chromatographic conditions according to Van Den Dool method [27].

### 2.5. Identification of Compounds

The constituents of the essential oils were identified by calculation of their retention indices under temperature-programmed conditions for *n*-alkanes (C_6_–C_24_) and the oil on a DB-5 column under the same chromatographic conditions. Identification of individual compounds was made by comparison of their mass spectra with those of the internal reference mass spectra library or with authentic compounds and confirmed by comparison of their retention indices with authentic compounds or with those of reported in the literature [28]. For quantification purpose, relative area percentages obtained by FID were used without the use of correction factors.

### 2.6. Antioxidant Activity

#### 2.6.1. DPPH Assay

Radical-scavenging activities (RSA) of *T. kotschyanus*, *T. eriocalyx,* and *T. daenensis subsp lancifolius* essential oils and extracts were determined using a published DPPH radical-scavenging activity assay method [29] with minor modifications. Briefly, stock solutions (10 mg/mL each) of the essential oils, extracts and the synthetic standard antioxidant BHT were prepared in methanol. Dilutions are made to obtain concentrations ranging from 1 to 5 × 10^−10^ mg/mL. Diluted solutions (2 mL each) were mixed with 2 mL of freshly prepared 80 *μ*g/mL DPPH methanol solution and allowed to stand for 30 min in the dark at room temperature for any reaction to take place. Ultraviolet (UV) absorbencies of these solutions were recorded on a spectrometer at 517 nm using a blank containing the same concentration of oils or extracts or BHT without DPPH. Inhibition of free radical DPPH in percent (*I*%) was calculated as follows

(1) $$I\%=\frac{A_{\text{blank}}-A_{\text{sample}}}{A_{\text{blank}}}\times 100,$$

where *A*_blank_ is the absorbance of the control reaction (containing all reagents except the test compound) and *A*_Sample_ is the absorbance of the test compound. The sample concentration providing 50% inhibition (IC_50_) was calculated by plotting inhibition percentages against concentrations of the sample. All tests were carried out in triplicate and IC_50_ values were reported as means ± SD of triplicates.

#### 2.6.2. *β*-Carotene/Linoleic Acid Bleaching Assay

The method described by Miraliakbari and Shahidi [30] was used with slight modifications. A stock solution of *β*-carotene and linoleic acid was prepared with 0.5 mg of *β*-carotene in 1 mL chloroform, 25 *μ*L of linoleic, acid and 200 mg Tween 40. The chloroform was evaporated under vacuum and 100 mL of aerated distiled water was then added to the residue. The samples (2 g/L) were dissolved in DMSO and 350 *μ*L of each sample solution was added to 2.5 mL of the above mixture in test tubes. The test tubes were incubated in a hot water bath at 50°C for 2 h, together with two blanks, one contained the antioxidant BHT as a positive control, and the other contained the same volume of DMSO instead of the extracts. The test tube with BHT maintained its yellow colour during the incubation period. The absorbencies were measured at 470 nm on an ultraviolet spectrometer. Antioxidant activities (inhibition percentage, *I*%) of the samples were calculated using the following equation:

(2) $$I\%=\left(\frac{A_{\beta \text{-carotene}\;\text{after}\;2\;\text{h}\;\text{assay}}}{A_{\text{initial}\;\beta \text{-carotene}}}\right)\times 100,$$

where *A*_*β*-carotene after  2 h assay_ is the absorbance of *β*-carotene after 2 h assay remaining in the samples and *A*_initial *β*-carotene_ is the absorbance of *β*-carotene at the beginning of the experiments. All tests were carried out in triplicate and inhibition percentages were reported as means ± SD of triplicates.

### 2.7. Assay for Total Phenolics

Total phenolic constituents of the polar and nonpolar subfractions of methanol extracts of *T*. *kotschyanus*, *T*. *eriocalyx,* and *T*. *daenensis subsp lancifolius* were determined by literature methods involving Folin-Ciocalteu reagent and gallic acid standard [30]. Solutions of the extracts (0.1 mL each) containing 1000 *μ*g of the extracts were taken individually in volumetric flasks, 46 mL of distilled water and 1 mL Folin-Ciocalteu reagent were added, and the flasks were thoroughly shaken. After 3 min, 3 mL of 2% Na_2_CO_3_ solution were added and the mixtures were allowed to stand for 2 h with intermittent shaking. Absorbencies were measured at 760 nm. The same procedure was repeated for all the standard gallic acid solutions (0–1000 mg/0.1 mL) and a standard curve obtained with the following equation:

(3) Absorbance=0.0012×gallic acid (μg)+0.0033.

Total phenols of the extract, as gallic acid equivalents, was determined by using the absorbance of the extract measured at 760 nm as input to the standard curve and the equation. All tests were carried out in triplicate and phenolic contents as gallic acid equivalents were reported as means ± SD of triplicate determinations.

### 2.8. Total Flavonoid Content (TFC)

Total flavonoid content was determined using the Dowd method as adapted by Arvouet-Grand et al. [31]. Briefly, 1 mL of 2% aluminum trichloride (AlCl_3_) in methanol was mixed with the same volume of the extracts (2000 *μ*g). Absorption readings at 415 nm were taken after 10 min against a blank sample consisting of a 1 mL extract solution with 1 mL methanol without AlCl_3_. The concentrations of flavonoid compounds were calculated according to the following equation that was obtained from the standard quercetin graph:

(4) $$\text{Absorbance}-0.026\;\text{quercetin}\;\left(\mu \text{g}\right)-0.0060\;\left({\text{R}}^{2}:\;0.9977\right)$$

## 3. Results and Discussion

### 3.1. Chemical Composition of the Essential Oils

The oils isolated by hydrodistillation from the aerial parts of *T*. *kotschyanus*, *T*. *eriocalyx,* and *T*. *daenensis subsp lancifolius* were found to be yellowish green, yellow and violate liquids and yields 2.11%, 1.55%, and 2.00% (w/w), based on dry weights, respectively. The chemical composition of the oils can be seen in Table 1. The components are listed in order of their elution on the DB-5 column. Forty-four components were identified in the essential oil of *T*. *kotschyanus*, the major components were found to be thymol (39.7%), *γ*-terpinene (11.4%), carvacrol (7.6%), and p-cymene (5.4%). According to Sefidkon et al carvacrol (40.7%–61.2%), thymol (7.5%–26.9%), and *γ*-terpinene (3.7%–8.2%) reported to be the main constituents of the volatile oil of *T*. *kotschyanus* collected from the suburb of Tehran before, at the beginning and end of flowering, respectively [3]. Thymol (38.0%), carvacrol (14.2%) and 1,8-cineole (13.2%) were identified as the main compounds of the oil of *T*. *kotschyanus* collected from Dizin of Iran [4]. In 2003, Rasooli and Mirmostafa reported carvacrol (35.1%), thymol (26.6%), and *γ*-terpinene (7.8%) before flowering and carvacrol (22.7%), thymol (16.5%), myrcene (12.6%), thymoquinone (11.4%), nerol (6.1%), and *β*-caryophyllene (5.5%) at the flowering stage as the major constituents of the oil of *T*. *kotschyanus* collected from Damavand area of Iran [1]. Pulegone (18.7%), isomenthone (17.8%), thymol (14.9%), 1,8-cineole (9.0%), piperitenone (6.3%), and carvacrol (5.5%) were previously reported as the major components of the essential oil of *T*. *kotschyanus* from Mazandaran province of Iran [2]. Carvacrol (44.2%) was also identified as major component of the oil of *T*. *kotschyanus* var. *glabrescens* collected from Turkey [6]. 

Thirty-eight compounds were identified in the oil of *T*. *daenensis subsp lancifolius*. The main components of this oil were carvacrol (52.3%), thymol (16.4%), *γ*-terpinene (10.8%), and p-cymene (3.3%), while Sadjadi and Khatamsaz study show that thymol (73.9%) carvacrol (6.7%) are the main components of *T. daenensis subsp lancifolius* [9]. Also comparing the volatile compounds of *T. daenensis subsp*. *lancifolius* oil and data published on the oil composition of *T. daenensis subsp*. *daenensis* shows that there are some qualitative and quantitative differences between the two oils. These chemical differences can most probably be explained by the variability of the plant subspecies and the existence of different chemotypes [5, 10, 11].

Thirty-eight compounds were characterized in the oil of *T*. *eriocalyx*, with thymol (42.6%), carvacrol (32.3%), p-cymene (4.1%), and *γ*-terpinene (3.0%) as main constituents. Kalvandi et al. show that thymol (42.8% and 43.1%), linalool (11.1% and 4.0%), *γ*-terpinene (6.0% and 6.3%), 1,8-cineole (5.6% and 3.3%), borneol (3.4% and 4.9%), and *α*-terpineol (1.8% and 7.1%) are the main constituents of *T. eriocalyx* collected from Markazi province of Iran, before flowering and full flowering stage, respectively [7]. Also, thymol (66.3%) and 1-borneol (10.5%) are reported in *T. eriocalyx* collected from Lorestan province of Iran [8]. The absence of carvacrol as the main component of *T. eriocalyx* oil, as mentioned above, is one major differences between our research and previous researches. 

The results showed different compositions of the essential oils of these *Thymus* species. The main compounds of essential oils of *T. kotschyanus*, *T. eriocalyx,* and *T. daenensis subsp lancifolius* species of *Thymus* which have been previously studied are presented in Table 2. Although comparison between compounds obtained from this study, and other reports, shows some similarities, but there are considerable quantitative and qualitative differences between these samples. This comparison also revealed that there are considerable differences even within the two samples from two different locations of Iran. These variations in the essential oil composition might have arisen from several differences (climatic, seasonal, geographical, and geological).

The presence of thymol, carvacrol, and *γ*-terpinene as the main components of *Thymus* species in this study, are similar to most of other investigations on other *Thymus* species. Although the main components of the oils of many *Thymus* species is thymol or carvacrol, other compounds like geraniol (30.3%) in *T. marshalliana*, *β*-caryophyllene (14.3% and 22.4%) in *T. transcaucasicus* and *T. balcanus*, 1,8-cineole (42.2–50.4%) in *T. mastichina*, and *α*-pinene (11.1%) in *T. coriifolius* were found to be the principal components of the oils.

### 3.2. Antioxidant Activity

*In vitro* antioxidant tests are designed to mimic the oxidation-reduction reactions popularly occurring in the live biological systems and are used to estimate the antioxidant potentials of various chemical and biological samples. In this research, two most widely used such assays, namely, 2,2-diphenyl-1-picrylhydrazyl (DPPH) and *β*-carotene/linoleic acid tests, were applied to evaluate the antioxidant capacities of essential oils and methanol extracts of *Thymus* species, and the results are given in Table 3. 

#### 3.2.1. DPPH Assay

The DPPH-radical-scavenging activity of the essential oils and the methanolic extracts are shown in Table 3. Lower IC_50_ value indicates higher antioxidant activity. Among total essential oils and methanol extracts, polar subfraction of methanol extract of *T. daenensis subsp lancifolius* have the highest antioxidant activitiy (IC_50_ = 19.1 ± 0.1 *μ*g/mL) which is close to synthetic antioxidant BHT (18.2 ± 0.3 *μ*g/mL). The DPPH-radical-scavenging activity of the oils, extracts, and BHT decreased in the order of BHT > polar subfraction of methanol extract of *T. daenensis subsp lancifolius* > polar subfraction of methanol extract of *T. eriocalyx*  > essential oil of *T*. *daenensis subsp lancifolius* > polar subfraction of methanol extract of *T. kotschyanus* > essential oil of *T*. *eriocalyx* > nonpolar subfraction of methanol extract of *T. daenensis subsp lancifolius* > essential oil of *T*. *kotschyanus* > nonpolar subfraction of methanol extract of *T. eriocalyx* > nonpolar subfraction of methanol extract of *T. kotschyanus*.

In the free-radical-scavenging activity, superiority of the polar subfraction of methanol extracts could be attributed to more amount of phenolic and flavonoid compounds [8]. The previous studies show that a few *Thymus* species could be proposed as very interesting natural resources with antioxidant activity such as *T. tosevii* var. *tosevii* (Kicevo), *T. tosevii* var. *degenii* (Kitka), *T. tosevii* var. *longifrons* (Kitka) *T. tosevii* ssp. substriatus (Kavadarci), and *T. longidens* var. *lanicaulis* (Sonje) [32]. Based on the results of this study *T. daenensis subsp lancifolius* can also be added to the above mentioned species which have antioxidant activity.

#### 3.2.2. *β*-Carotene Bleaching Test

The results of essential oils, methanol extract samples and BHT antioxidant activity are presented in Table 3. Both the essential oils and methanol extracts obtained from *Thymus* species prevented the bleaching *β*-carotene. As shown in Table 3, the % inhibition capacity of the polar subfraction leaves methanol extract (96.1 ± 0.8) was found to be superior to all samples, which is almost equal to the inhibition capacity of positive control BHT (96.4% ± 0.9). In *β*-carotene/linoleic acid assay, *T. eriocalyx* nonpolar subfraction (96.1% ± 0.8), *T. daenensis subsp lancifolius* nonpolar subfraction (92.1% ± 1.1) *T. daenensis subsp lancifolius* oil (88.4% ± 0.8) have also notable antioxidant activity.

The antioxidant activity may be due to different mechanisms, such as prevention of chain initiation, decomposition of peroxides, prevention of continued hydrogen abstraction, free-radical scavenging, reducing capacity, and binding of transition metal ion catalysts [32]. Compared to reported essential oil compositions of different *Thymus* species, investigations on their biological activities are still scarce, because the area of investigation on biological activities of different *Thymus* species is very abundant.

### 3.3. Assays for Total Phenolics and Flavonoids

Based on the absorbance values of the methanolic extracts, reacting with Folin-Ciocalteu reagent and compared with the standard solutions of gallic acid equivalents, as described above, results of the colorimetric analysis of total phenolics are given in Table 4. The amount of total phenolics in the polar extracts of *T. eriocalyx*, *T. daenensis subsp lancifolius,* and *T. kotschyanus* were 244.8 ± 2.2 *μ*g GEs/mg extract (24.4%, w/w), 147.2 ± 1.2 *μ*g GEs/mg extract (14.7%, w/w) and 117 ± 0.9 *μ*g GEs/mg extract (11.7%, w/w) which the values of *T. daenensis subsp lancifolius* and *T. kotschyanus* are comparable to the values reported in the literature for other *Thymus* species such as *Thymus spathulifolius* (141 *μ*g/mg) of the polar subfraction of a methanol extract, reported by Sokmen et al. (2004)) [32], *Thymus caramanicus* (12.4 *μ*g/mg) of the polar subfraction of a methanol extract, reported by Safaei-Ghomi et al. (2009) and *Thymus Serpyllum* (113 *μ*g/mg) of an ethanol extract, reported by Matta et al. (2007) [33], while phenolic compounds in the polar subfraction of a methanol extract of *T. eriocalyx* (244.8 ± 2.2 *μ*g/mg) is more than reported *Thymus* species.

The total flavonoid contents obtained from methanol extracts of *Thymus* species were ranked as follows: polar subfraction of *T. daenensis subsp lancifolius* > polar subfraction of *T. kotschyanus* > polar subfraction of *T. eriocalyx* > nonpolar subfraction of *T. daenensis subsp lancifolius* > nonpolar subfraction of *T. eriocalyx* > nonpolar subfraction of *T. kotschyanus* (Table 4).

## 4. Conclusion

*Thymus* species are well known as aromatic and medicinal plants because of their biological and pharmacological properties. The antioxidant activity of basil, oregano, and thyme essential oils has been evaluated in a series of *in vitro* tests [15]. The oils of the three investigated species are rich in monoterpene phenols (especially, thymol and carvacrol) and due to this high phenol content, they can be considered as substitutes for *Thymus vulgaris* (common thyme) oil for medicinal purposes and other applications. 

In the present study, in some cases, essential oils and methanol extracts showed similar or near to similar antioxidant activities compared to the standard BHT. Nowadays, there is great worldwide interest in finding new and safe antioxidants from natural sources to prevent oxidative deterioration of foods and to minimise oxidative damage of living cells. Within *Lamiaceae* species examples of new antioxidants include: phenolic diterpenes, phenolic carboxylic acids, biphenyls, and flavonoids extracted from rosemary, sage, oregano, and thyme [34].

## Figures and Tables

**Table 1:** Chemical composition of essential oils of *Thymus* species.

No.	Compound	RI	*T. kotschyanus*	*T. daenensis subsp lancifolius*	*T. eriocalyx*
(1)	tricyclene	921	0.1	—	—
(2)	*α*-thujene	928	0.5	0.9	0.4
(3)	*α*-pinene	935	0.5	0.4	1.6
(4)	camphene	947	—	0.2	0.7
(5)	sabinene	970	0.1	0.1	0.1
(6)	*β*-pinene	974	0.2	0.1	0.3
(7)	3-octanone	975	0.2	0.6	0.1
(8)	1-octen-3-ol	978	0.1	0.1	—
(9)	*β*-myrcene	988	0.7	1.2	0.8
(10)	3-octanol	993	0.1	0.1	—
(11)	*α*-phellandrene	1003	0.1	0.2	0.1
(12)	*δ*-3-carene	1008	—	—	0.1
(13)	*α*-terpinene	1013	1.2	1.2	0.9
(14)	p-cymene	1024	5.4	3.3	4.1
(15)	limonene	1027	0.2	0.2	0.3
(16)	1,8-cineol	1031	1.4	0.6	0.8
(17)	cis-ocimene	1038	0.1	—	—
(18)	trans-ocimene	1048	0.7	0.1	—
(19)	*γ*-terpinene	1057	11.4	10.8	3
(20)	trans-sabinene hydrate	1064	—	—	0.1
(21)	terpinolene	1087	0.2	0.1	0.2
(22)	cis-sabinene hydrate	1097	2.9	0.3	0.1
(23)	linalool	1099	0.1	0.3	0.4
(24)	camphor	1143	0.1	—	0.1
(25)	borneol	1166	4.4	0.7	2.1
(26)	terpinene-4-ol	1179	2.5	0.5	1.7
(27)	*β*-fenchyl alcohol	1183	0.2	—	—
(28)	dihydrocarvone	1186	—	0.2	—
(29)	*α*-terpineol	1192	0.3	0.2	—
(30)	carvacrol methyl ether	1214	—	0.1	0.4
(31)	pulegone	1218	—	—	1.1
(32)	citral	1233	0.3	—	—
(33)	geraniol	1255	0.2	—	—
(34)	thymol	1278	39.7	16.4	42.6
(35)	thymol acetate	1285	—	0.1	—
(36)	carvacrol	1291	7.6	52.3	32.3
(37)	bornyl acetate	1303	0.2	—	0.4
(38)	piperitenone	1315	—	—	0.4
(39)	geranyl acetate	1362	0.1	—	—
(40)	*β*-bourbonene	1382	0.1	—	0.1
(41)	*β*-elemene	1388	0.1	—	—
(42)	*α*-gurjunene	14.6	1.7	—	—
(43)	*β*-caryophyllene	1414	—	1.8	1.7
(44)	allo-aromadendrene	1458	—	0.1	0.2
(45)	germacrene-D	1478	1.3	0.4	0.1
(46)	valencene	1491	0.1	—	0.1
(47)	ledene	1483	0.1	—	—
(48)	bicyclogermacrene	1488	0.3	0.3	—
(49)	*β*-bisabolene	1511	0.1	0.6	0.4
(50)	*β*-cadinene	1518	0.1	—	—
(51)	*δ*-cadinene	1526	—	0.1	0.2
(52)	*γ*-bisabolene	1533	1.2	0.7	0.8
(53)	veridiflorol	1608	—	0.1	—
(54)	spathulenol	1577	0.3	0.2	0.4
(55)	caryophyllene oxide	1581	0.6	0.2	0.5
(56)	*γ*-gurjunene		2.2	—	—

**Table 2:** The chemical composition of essential oil of studied *Thymus* species.

Species	Components	References
*T. kotschyanus *	carvacrol (35.1%), thymol (26.6%), before flowering and carvacrol (22.7%), thymol (16.5%), myrcene (12.6%), thymoquinone (11.4%) at the flowering stage	Rasooli and Mirmostafa [1]
*T. kotschyanus *	pulegone (18.7%), isomenthone (17.8%), thymol (14.9%),	Morteza-Semnani et al. [2]
*T. kotschyanus *	carvacrol (40.7%–61.2%), thymol (7.5%–26.9%), *γ*-terpinene (3.7%– 8.2%) at the beginning of and at the end of flowering stages, respectively	Sefidkon et al. [3]
*T. kotschyanus *	thymol (37.9%), carvacrol (14.1%)	Rustaiyan et al. [4]
*T. kotschyanus *	thymol (38.6%), carvacrol (33.9%)	Nickavar [5]
*T. kotschyanus *	carvacrol (44.2%)	Mericli [6]
*T. eriocalyx *	thymol (42.8%) before flowering	Kalvandi [7]
*T. eriocalyx *	thymol (43.1%) at full flowering	Kalvandi [7]
*T. eriocalyx *	thymol (66.3%), 1-borneol (10.5%)	Sfaei-Ghomi et al. [8]
*T. daenensis subsp. lancifolius *	thymol (73.9%), carvacrol (6.7%)	Sajjadi and Khatamsaz [9]
*Thymus daenensis subsp. daenensis*	thymol (74.7%), p-cymene (6.5%)	Nickavar [5]
*Thymus daenensis subsp. daenensis*	geraniol (34.9%–37.2%), geranyl acetate (15.3%–18.7%), geranial (9.0%–11.2%)	Hashemi et al. [10]
*Thymus daenensis*	thymol (51.3%), p-cymene (2.7%–7.6%)	Barazandeh and Bagherzadeh [11]

**Table 3:** Antioxidative capacities of the essential oils and methanol extracts of *Thymus* species ^*a*^.

Plant oils, methanol extracts,and controls	Test system	
	DPPH IC_50_ (*μ*g/mL)	*β*-Carotene/linoleic acid (% inhibition rate)
*T. kotschyanus* oil	278 ± 1.9	69.2 ± 0.5
*T. daenensis subsp lancifolius* oil	99.6 ± 0.5	88.4 ± 0.8
*T. eriocalyx* oil	198 ± 1.4	57.6 ± 0.6
*T. kotschyanus* polar sub-fraction	128 ± 0.9	61.2 ± 0.5
*T. kotschyanus* non-polar sub-fraction	526 ± 3.1	81.4 ± 0.6
*T. daenensis subsp lancifolius* polar sub-fraction	19.1 ± 0.1	50.2 ± 0.4
*T. daenensis subsp lancifolius* non-polar sub-fraction	248.7 ± 1.6	92.1 ± 1.1
*T. eriocalyx* polar sub-fraction	34.2 ± 0.4	67.9 ± 0.7
*T. eriocalyx* non-polar sub-fraction	426.2 ± 2.7	96.1 ± 0.8
BHT	18.2 ± 0.3	96.4 ± 0.9

^a^Results are means of three different experiments.

**Table 4:** Amount of total phenolics and flavonoids in the methanol extracts of *Thymus* species ^*a*^.

Sample	Phenolic content (*μ*g GEs/mg extract)^b^	Flavonoid content (*μ*g QEs/mg extract)^c^
*T. kotschyanus* polar sub-fraction	117 ± 0.9	36.7 ± 0.7
*T. kotschyanus* non-polar sub-fraction	41.1 ± 0.3	8.7 ± 0.2
*T. daenensis subsp lancifolius* polar sub-fraction	147.2 ± 1.2	48.8 ± 0.7
*T. daenensis subsp lancifolius* non-polar sub-fraction	32.8 ± 0.3	16.4 ± 0.5
*T. eriocalyx* polar sub-fraction	244.8 ± 2.2	25.5 ± 0.4
*T. eriocalyx* non-polar sub-fraction	62.5 ± 0.6	13.2 ± 0.3

^a^results  are means of three different experiments.

^b^GEs, Gallic acid equivalents.

^c^QEs: quercetin equivalents.
